# Optimization of Gelatin-Based Scaffolds for Soft Tissue Regeneration: In Vitro and In Vivo Performance

**DOI:** 10.3390/ijms26189106

**Published:** 2025-09-18

**Authors:** Zita Szűcs-Takács, Viktória Varga, Fanni Bán, Viktória Harcsa, Balázs Pinke, Róbert Várdai, Fatime Gajnut, Enikő Major, István Hornyák

**Affiliations:** 1 Institute of Translational Medicine, Semmelweis University, 1094 Budapest, Hungary; takacs.zita@semmelweis.hu (Z.S.-T.); varga.viktoria.dora@phd.semmelweis.hu (V.V.); fanni339@gmail.com (F.B.); harcsaviki99@gmail.com (V.H.); gajnutfatime@gmail.com (F.G.); major.eniko@semmelweis.hu (E.M.); 2 Department of Polymer Engineering, Faculty of Mechanical Engineering, Budapest University of Technology and Economics, 1111 Budapest, Hungary; pinke@pt.bme.hu; 3 Laboratory of Plastics and Rubber Technology, Department of Physical Chemistry and Materials Science, Faculty of Chemical Technology and Biotechnology, Budapest University of Technology and Economics, 1111 Budapest, Hungary; vardai.robert@vbk.bme.hu; 4 Research Centre for Natural Sciences, Institute of Materials and Environmental Chemistry, 1111 Budapest, Hungary; 5 HUN-REN-SU Cerebrovascular and Neurocognitive Disease Research Group, 1094 Budapest, Hungary

**Keywords:** cross-linked gelatin, soft tissue implant, biomaterials, in vivo remodeling

## Abstract

In this study, promising compositions of cross-linked gelatin-based scaffolds were tested in vitro and in vivo. Our aim was to utilize a solid matrix that is suitable for medical applications, and to be regulated as a medical device as a soft tissue implant. Three different cross-linkers were used in vitro, and the optimal composition was chosen for in vivo testing. The surfaces of the scaffolds were observed with SEM, and, in the case of divinyl sulfone (DVS), small cracks appeared, and the structure was rigid. With the use of poly(ethylene glycol) diglycidyl ether (PEGDE), the surface was found to be uneven, but generally, the appearance was similar in each case. The optimal scaffold contained 5 *v*/*v* % 1,4-butanediol diglycidyl ether (BDDE), and was implanted for either one month or three months in the back of BL6 mice. The explants were assessed using analytical techniques, including microscopic imaging and histological analysis, and it was found that cells, connective tissue, and extracellular matrix (ECM) were all able to successfully infiltrate the scaffolds and did not induce any inflammation. In summary, these novel implants seem to promote blood vessel formation and support the adherence of adipose tissue, as confirmed by optical microscopy and histological evaluations.

## 1. Introduction

Tissue engineering (TE), originally defined as the development of functional substitutes for damaged tissues [1], has progressively developed in the past decades, due to numerous types of biomaterials and replacement solutions [2,3,4]. TE is an interdisciplinary field that combines three major disciplines: materials science, engineering, and biology. One of the main scopes of TE is the development and preparation of scaffolds [5]. Generally, these materials are intended to be biocompatible, and among these materials, the aim can be to prepare a permanent implant for soft tissue regeneration [6]. These scaffolds are typically designed to serve as artificial support and to create a conducive environment for the attachment of cells and the formation of tissues [7]. Scaffolds can exist in various states, such as liquid, gel-like, or solid [8]. Currently, 3D printing (3DP) is receiving significant attention in the field of TE. Among the suitable biomaterials, polylactic acid (PLA) and polycaprolactone (PCL) stand out due to their remarkable physicochemical characteristics and biocompatibility, making them highly appealing for tissue regeneration applications. But in every case, it is vital to be compatible with the surrounding tissues and be suitable for medical applications [9].

Scaffold materials can be prepared using natural or synthetic polymers and can be designed to either degrade over time or remain non-degradable, depending on the intended application. Biodegradable synthetic polymers suitable for implantation offer a wide variety of macromolecules; however, the most generally applied are PLA and PCL [10]. Nevertheless, synthetic polymers generally exhibit lower cell affinity compared to natural polymers. While PCL has the benefits such as biodegradability and biocompatibility, its limitations include reduced hydrophobicity, slower degradation rate, and the absence of surface cell recognition sites [11]. PCL appears in many forms, from films, matrices, membranes, to capsules, fibers. PLA scaffolds are known for their excellent mechanical and thermal properties [12]. Additionally, these materials must be biocompatible, meaning they should not be cytotoxic or trigger significant immune responses, according to the applicable standards [6,13], should withstand sterilization [14], and have harmless degradation products [15]. Synthetic scaffolds offer significant advantages due to their highly customizable characteristics, such as porosity, degradation, and mechanical strength [7]. On the other hand, natural scaffolds are often preferred for their well-known and documented interactions and minimal immune response [16]. For soft tissue applications, the pore size and relatively high surface of the material are important characteristics to support cell growth and tissue formation [8]. Regardless of what kind of material is used, the fabrication method also plays a major role in TE. Scaffold features like pore size, shape, and interconnectivity directly affect how cells migrate and proliferate [11]. The most widely known and used fabrication techniques are solvent casting, gas foaming, porogen leaching, thermally induced phase separation (TIPS), and freeze drying. Three-dimensional printing and electrospinning are advanced methods of scaffold manufacturing. Electrospinning is a fabrication method that employs an electric field to draw and spin a polymer solution into nanofibers deposited on a substrate. This technique is highly regarded in TE due to its ability to produce structures with a high-surface-area-to-volume ratio, enhancing cell attachment and nutrient exchange [17]. However, since the freeze-drying method is less expensive and is more standardized for water-soluble materials, we chose this method for our set of experiments.

The study aims to investigate a solid matrix that is suitable for long-term implantation as a soft tissue implant. To obtain a material that closely mimics soft tissue, we selected a material that is derived from collagen.

Collagen is among the most extensively studied and widely available natural biomaterials [18,19,20], whereas this material forms a part of the extracellular matrix in most connective tissues within mammals [21].

Due to its biocompatibility, biodegradability, and broad availability, it is an appealing option for a range of biomedical applications through chemical modifications, to create hydrogels, membranes, sponges, and scaffolds [14,18]. Following modification with multifunctional biological molecules, biocompatible polymers, and other bioactive substances, collagen is extensively applied in TE disciplines, including bone and cartilage, skin, neural, and tendon TE [22]. Collagen has the ability to interact with cell surface receptors, thus promoting cell adhesion, proliferation, migration, and differentiation, which are essential for wound healing and tissue regeneration [23,24]. Since it has a fibrillar structure, collagen is crucial for preserving the structural integrity and functionality of the extracellular matrix, thereby offering essential mechanical support to tissues [25].

Beyond proliferation, collagen also plays a beneficial role in other stages of wound healing, including hemostasis, inflammation, and tissue remodeling [26]. Another interesting aspect of collagen is that it can be cross-linked, partially degraded, and modified to prepare new materials [27], which was our approach as well for this study.

Gelatin is a natural polymer derived from the partial hydrolysis of insoluble native collagen [28]. Gelatin’s characteristics are similar to collagen and can be metabolized by human tissues without triggering an immune response [29]. Due to its production through collagen hydrolysis, gelatin dissolves in water at physiological temperatures and forms a gel-like structure, which means that the main disadvantage of gelatin is the low melting point of its aqueous solution [30]. Generally, mammalian gelatin has a higher melting point (~33 °C) compared to fish-derived gelatin [31]. Our choice was porcine collagen-derived gelatin. In order to enhance mechanical properties, various cross-linking methods can be used, and/or functional groups can be modified [8]. Cross-linking has the potential to improve the mechanical properties and stability of the composite, thereby extending its functional lifespan in the body and enhancing both the efficacy and durability of the material [32].

Epoxy compounds have functional groups capable of reacting with amino, carboxyl, and hydroxyl groups, making them highly effective for cross-linking [33,34]. In the study, three different cross-linkers were used: 1,4-butanediol diglycidyl ether (BDDE), poly(ethylene glycol) diglycidyl ether (PEGDE), and divinyl sulfone (DVS). These cross-linking methods transform gelatin into a solid form. In our previous work, BDDE was investigated and generally found safe to use due to its good biocompatibility, and is suitable to cross-link primary amino groups. PEGDE also has the potential to be used as a cross-linker, as the structure is similar to BDDE, although PEGDE has higher hydrophilicity and a longer molecular chain [35]. Vinyl sulfone derivatives are recognized as efficient cross-linking agents in the design of biomaterials [36]. In this study, we also utilized divinyl sulfone (DVS), which has demonstrated effectiveness in our previous experiments involving hyaluronic acid as the starting material. The objective of the study was to use a novel approach that involves the use of solid freeze-dried gelatin-based sponge-like scaffolds embedded within a cross-linker matrix, focusing on optimizing the reaction parameters and evaluating the properties of the cross-linked matrices, in vitro and in vivo.

## 2. Results

### 2.1. Scaffold Structure

**Figure 1:**
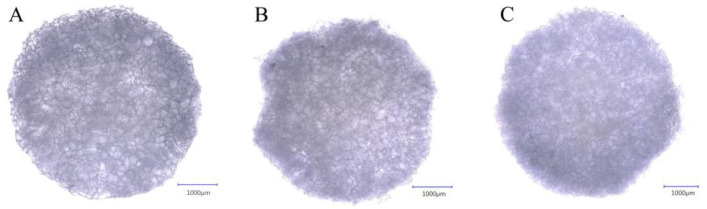
The appearance of the cross-linked matrices with the use of a digital optical microscope (Keyence VHX-5000, VH-Z100R objective, 100× magnification) 50G5B (**A**), 50G5D (**B**), 50G5P (**C**), from left to right. The matrices were 5 mm in diameter and were 2 mm thick. The matrices were similar in appearance and generally sponge-like.

The cross-linked matrices were washed and sterilized using heat sterilization, and cut with 5 mm diameter biopsy punches. The matrices were freeze-dried under aseptic conditions to reach the form that was tested in vitro and in vivo. The physical appearance of the scaffolds can be seen below. As it is visible in Figure 1, the dry matrices retained their shape and the porous structure.

**Figure 2:**
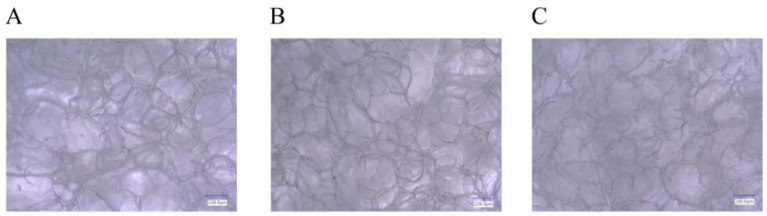
Sponge-like structure of the scaffold with the use of a digital optical microscope (Keyence VHX-5000, VH-Z100R objective, 300× magnification) 50G5B (**A**), 50G5D (**B**), 50G5P (**C**), from left to right.

The inner structure was further visualized using higher magnification, which is presented in Figure 2.

### 2.2. Cell-Viability Experiment

**Figure 3:**
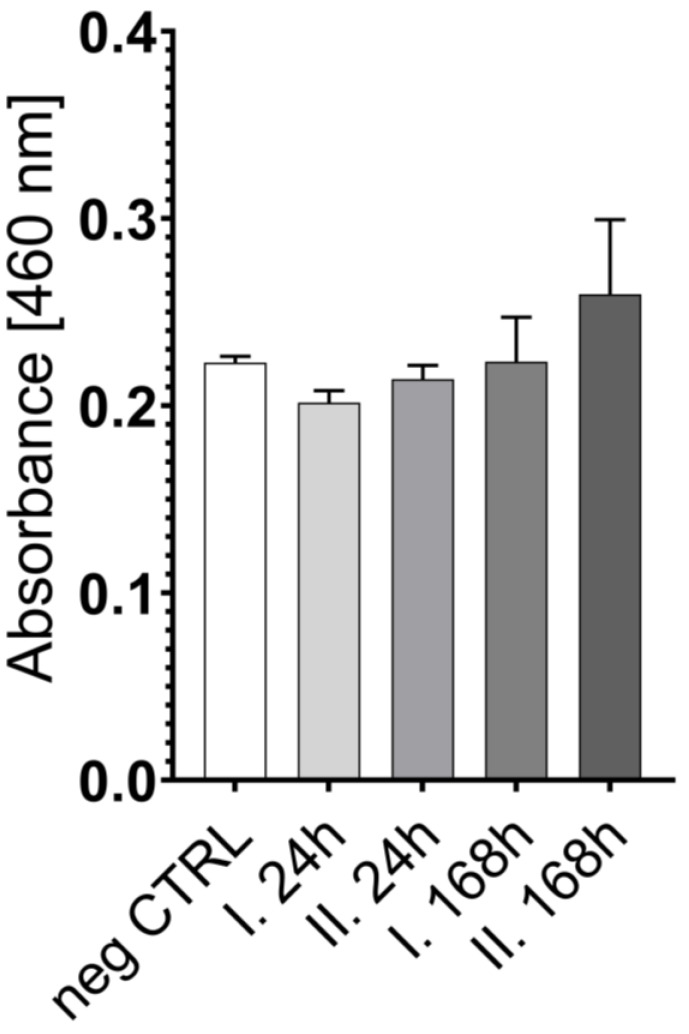
The cell attachment and proliferation on the two optimal scaffolds using hBM-dMSCSs. The absorbances were measured after 24 and 168 h of culturing: 5 *w*/*w* % GEL, cross-linked in 20 µL BDDE and 300 µL 1 *w*/*w* % NaOH (I), 5% GEL, cross-linked in 40 µL BDDE and 600 µL 1 *w*/*w* % NaOH (II).

Based on the structural and mechanical testing in our earlier work, we chose two matrices that were found to be optimal to be tested in vitro and in vivo [8]. The scaffolds were used in cell culture to evaluate if they support the attachment and proliferation of the cells on 5 mm diameter scaffolds. XTT measurements were performed to investigate cell proliferation after 24 h and 168 h using Cell Proliferation Kit II (XTT; Roche, Mannheim, Germany). The absorbance of the scaffolds at 460 nm is shown in Figure 3.

**Figure 4:**
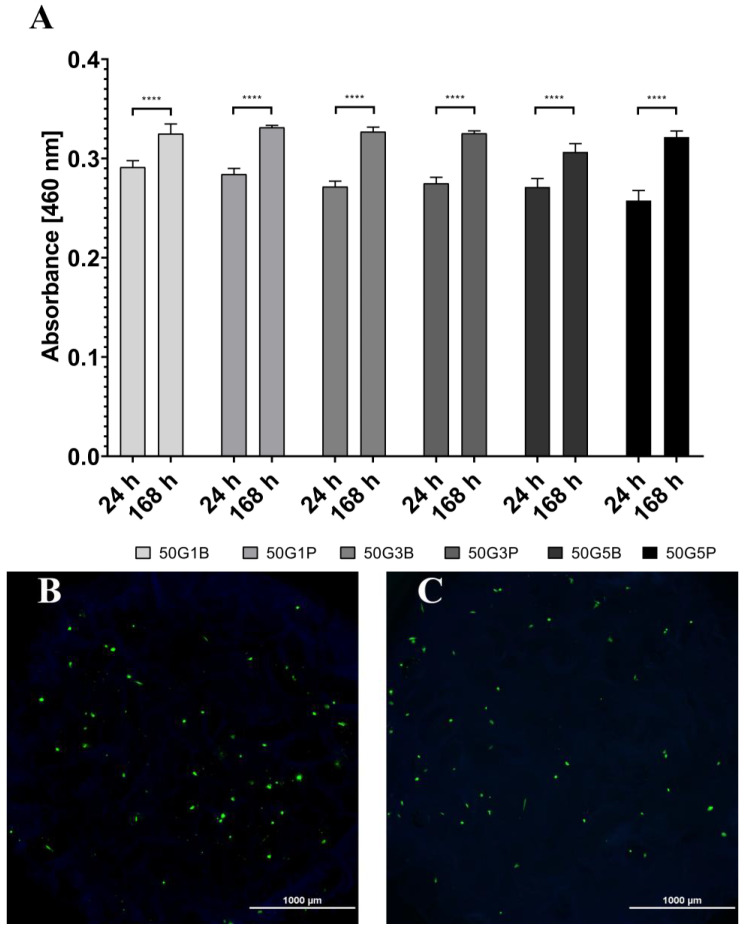
Cell viability measurement after attachment (24 h) and after 168 h of incubation on the 2 cm diameter scaffolds prepared using three BDDE (50G1B, 50G3B, 50G5B) and PEGDE (50G1P, 50G3P, 50G5P) cross-linker concentrations (1, 3, 5 *v*/*v* %) (**A**). Representative fluorescent microscopic images of 5 mm diameter scaffolds prepared from the original 2 cm diameter scaffolds using a biopsy punch, 4× magnification 50G1B (**B**) and 4× magnification 50G5P (**C**). **** *p* < 0.0001.

According to the results, there was no significant difference between the viability after 24 h and 168 h on the two optimal matrices. Thus, reduced BDDE cross-linker concentration was tested with XTT on 2 cm diameter matrices to see the potential effect on cell attachment and proliferation; additionally, PEGDE was also tested in 5 *v*/*v* % concentration. Representative microscopic images were also taken of 5 mm diameter matrices; these results are depicted in Figure 4.

**Figure 5:**
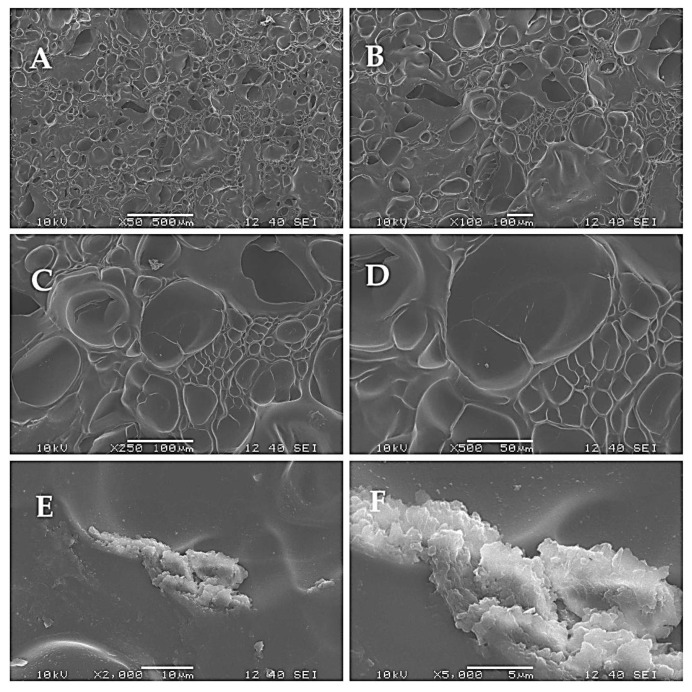
Surface of the scaffold using BDDE as a cross-linker: (**A**) 50×, (**B**) 100×, (**C**) 250×, (**D**) 500×, (**E**) 2000×, and (**F**) 5000× magnification.

**Figure 6:**
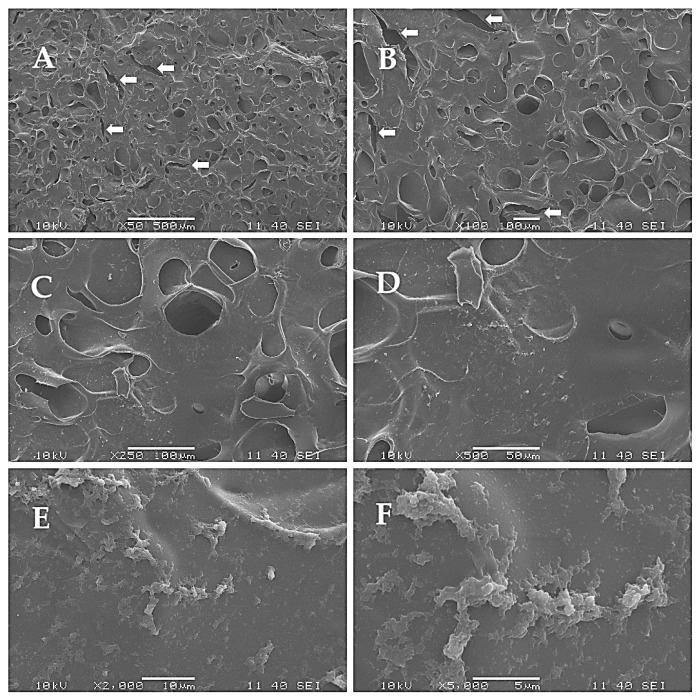
Surface of the scaffold using DVS as a cross-linker. Small cracks are visible on the surface of the matrix, indicated with white arrows: (**A**) 50×, (**B**) 100×, (**C**) 250×, (**D**) 500×, (**E**) 2000×, and (**F**) 5000× magnification.

**Figure 7:**
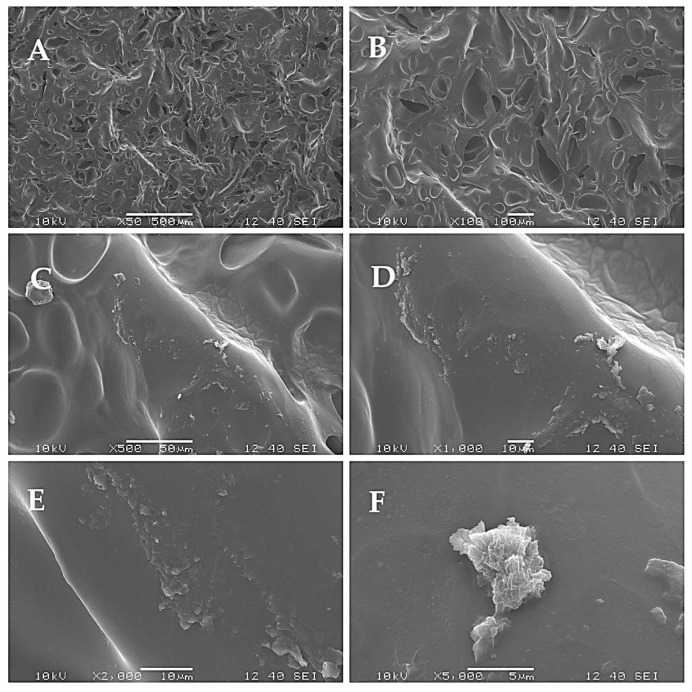
Surface of the scaffold using PEGDE as a cross-linker: (**A**) 50×, (**B**) 100×, (**C**) 500×, (**D**) 1000×, (**E**) 2000×, and (**F**) 5000× magnification.

All scaffolds exhibited relatively similar absorbance values both after 24 h and 168 h of culturing, with significant differences observed between the 24 h results and the 168 h results in all the groups. This indicates that all the scaffolds were suitable to support the viability of the cells. The differences between the surfaces were also compared using SEM. In this case, BDDE, DVS, and PEGDE were used as cross-linkers at a concentration of 5 *v*/*v* %. Cells were seeded on the matrices and were fixed after one week of culturing. The surfaces of the samples are visible in Figure 5, Figure 6 and Figure 7.

### 2.3. SEM Image Analyses

**Figure 8:**
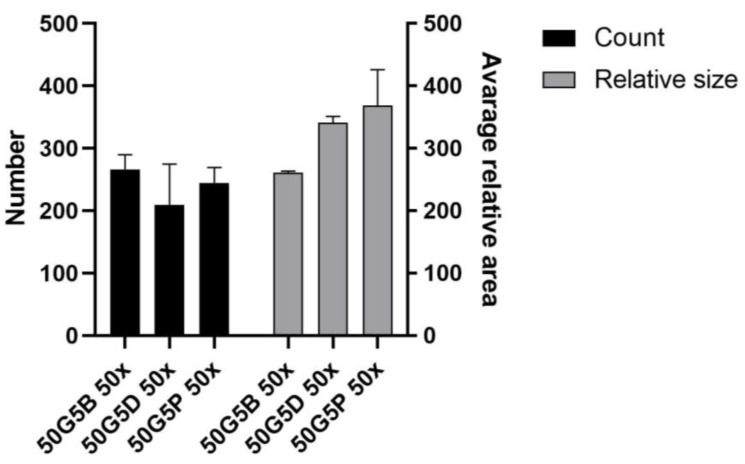
Number of pores and average relative area (pixels) of the pores using the three different cross-linkers. The calculation was performed using ImageJ using the 50× magnification images.

Generally, the surface was similar in all cases; one major difference can be seen as a difference between the epoxy and vinyl cross-linkers. In the case of DVS, small cracks are visible on the surface of the matrix, indicated with white arrows. For a better understanding, the number of pores and the relative pore sizes were compared using ImageJ 1.53o, and the results are shown in Figure 8.

As it is visible in the SEM images and was quantified in Figure 8, the number of pores was similar with the use of all three cross-linkers; however, the relative mean area of an average pore was the lowest in the case of BDDE, albeit the difference was not significant.

Thus, based on our previous experiments and the in vitro results, the conclusion was that the scaffolds can support cell attachment, which indicates that these were sterile, non-toxic, and had similar pore sizes. The main purpose of the development was to fabricate a material that is suitable for implantation for a longer period of time. Thus, given the fragile structure of the matrices prepared with DVS and the rather uneven surface of PEGDE, we chose the matrices that were originally considered to be optimal according to the results of the mechanical experiments [8]. Therefore, the 5 V/V % BDDE matrices were chosen for the in vivo experiments, and were implanted for either one month or three months in the back of BL6 mice.

**Figure 9:**
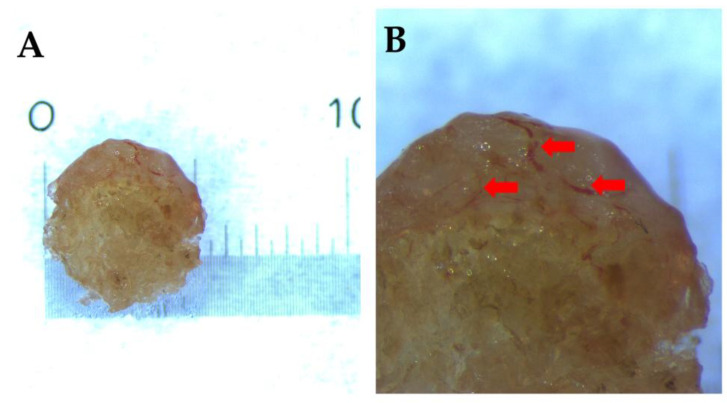
Microscopic images of the explanted scaffold after 1 month. (**A**) shows the actual size of the implant, which was ~5 mm as seen on the scale. (**B**) highlights the integration of vascular structures (indicated with red arrows).

**Figure 10:**
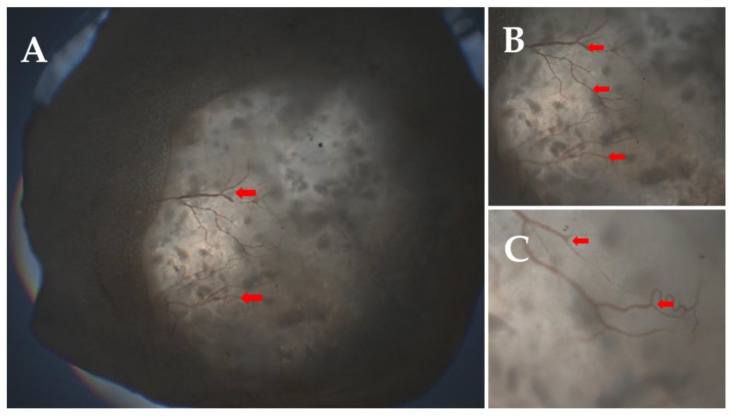
Newly formed blood vessels after 1 month (indicated with red arrows): 2× (**A**), 4× (**B**), and 10× (**C**) magnification.

After explantation, the matrices were examined using light microscopy and histology. The explanted materials did not show any sign of infection, and, generally, live tissue was starting to infiltrate the matrices. Both fibrous tissue on the outside and blood vessel formation in the inside were visible, as shown in the demonstrative images, Figure 9 and Figure 10.

### 2.4. Microscopic Images of the Explant

**Figure 11:**
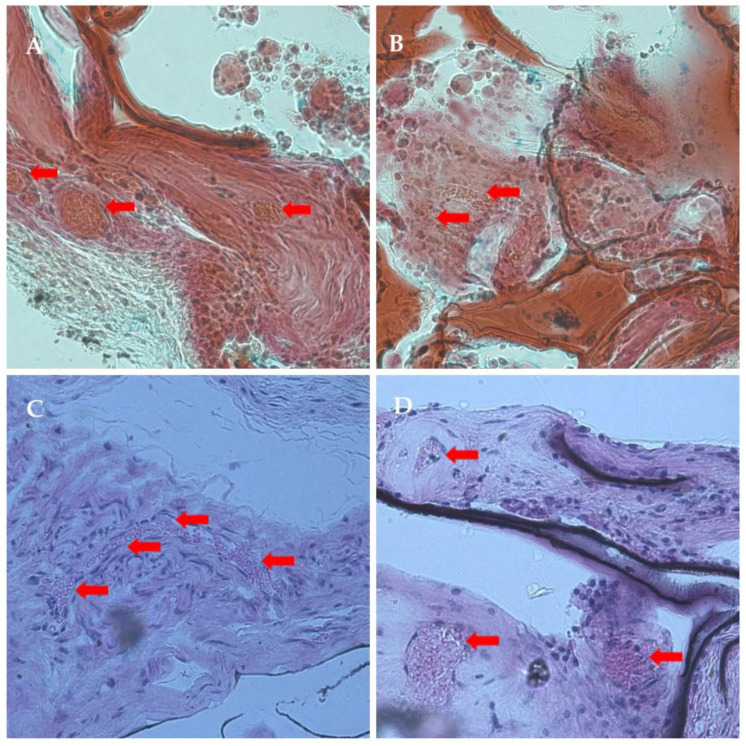
Histological images of the explants. Different tissue staining methods were used on the samples (**A**,**B**): Movat’s pentachrome staining were used to demonstrate collagen and reticular fibers (yellow), nuclei and elastic fibers (black/blue), and fibrin and muscle (red); (**C**,**D**) Hematoxylin–Eosin staining were used to localize extracellular matrix features (pink) and cell nuclei (blue/purple). The presence of red blood cells and newly formed blood vessels is indicated with red arrows.

After explantation, the samples were dehydrated and embedded in MMA (methyl methacrylate), and stained for further examination. The main findings were that cells, connective tissue, and ECM were able to infiltrate the scaffolds, as visible in the images below. The formation of blood vessels was also visible; the vessels are indicated with red arrows in Figure 11.

**Figure 12:**
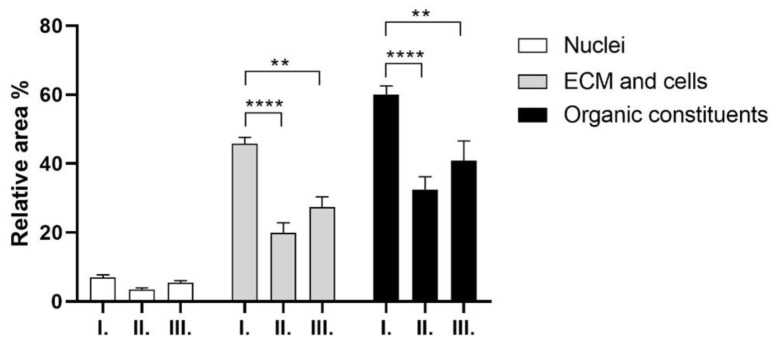
Elastica van Gieson (EvG) staining of the explants: 5 *w*/*w* % GEL, cross-linked in 20 µL BDDE and 300 µL 1 *w*/*w* % NaOH for 4 weeks (I), 5 *w*/*w* % GEL, cross-linked in 40 µL BDDE and 600 µL 1 *w*/*w*% NaOH for 4 weeks (II), 5 *w*/*w* % GEL, cross-linked in 20 µL BDDE and 300 µL 1 *w*/*w* % NaOH for 12 weeks (III), (n = 4). ** *p* < 0.01 and **** *p* < 0.0001.

**Figure 13:**
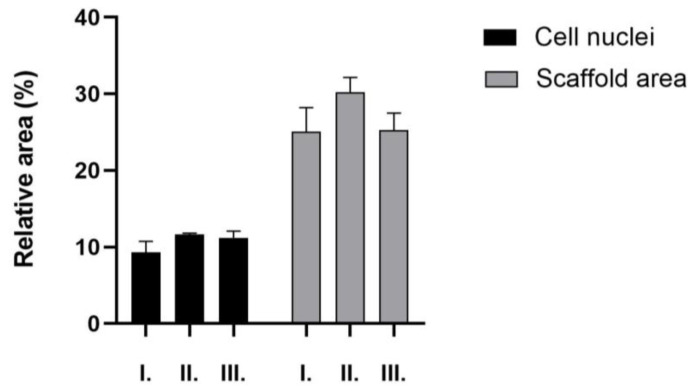
Hematoxylin and Eosin (H&E) staining of the explants: 5 *w*/*w* % GEL, cross-linked in 20 µL BDDE and 300 µL 1 *w*/*w* % NaOH for 4 weeks (I), 5 *w*/*w* % GEL, cross-linked in 40 µL BDDE and 600 µL 1 *w*/*w* % NaOH for 4 weeks (II), 5 *w*/*w* % GEL, cross-linked in 20 µL BDDE and 300 µL 1 *w*/*w* % NaOH for 12 weeks (III), (n = 3).

**Figure 14:**
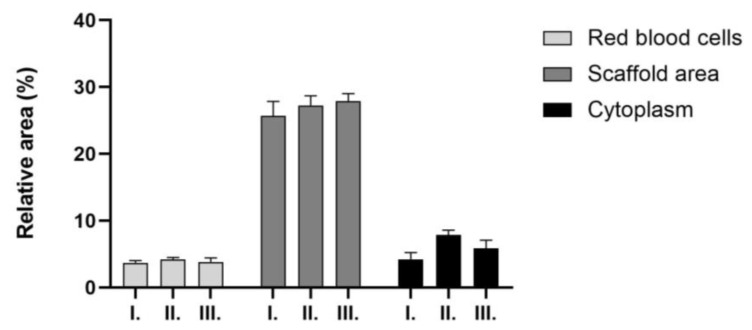
Masson and Goldner (MG) staining of the explants: 5 *w*/*w*% GEL, cross-linked in 20 µL BDDE and 300 µL 1 *w*/*w* % NaOH for 4 weeks (I), 5 *w*/*w* % GEL, cross-linked in 40 µL BDDE and 600 µL 1 *w*/*w* % NaOH for 4 weeks (II), 5 *w*/*w* % GEL, cross-linked in 20 µL BDDE and 300 µL 1 *w*/*w* % NaOH for 12 weeks (III), (n = 3).

To quantify the contents of the slides after staining, ImageJ was used to characterize the relative areas of the specific constituents in Figure 12, Figure 13 and Figure 14.

According to the EvG staining, the area occupied by cell nuclei is relatively low across all three samples. There is no significant difference between the 4-week and 12-week samples. The lowest value is observed in sample “II”. The ratio of ECM and cells is the highest in sample “I”, which is significantly higher compared to samples “II” and “III”. H&E staining was also evaluated. With this method, the cells and the scaffold area can be quantified (Figure 13).

There was no significant difference between the area of the nuclei and the scaffold in either of the sample groups. MG staining was also tested to quantify the vessels; the results are shown in Figure 14.

The presence of red blood cells was similar in all the sample groups, as well as the area of the scaffold and the cytoplasm; there were no significant differences in the values in either group.

## 3. Discussion

Although the starting materials, gelatin and epoxy cross-linkers, are already well-known and well-described in the field, we managed to use gelatin in a freeze-dried form and were able to effectively cross-link it in a solid state, which was considered to be novel, inventive, and industrially applicable; thus, a patent application was filed and is now in the PCT phase. Our previous experiments led to the optimal parameters for cross-linking gelatin to suit our purposes and identifying the most important mechanical properties [8]. In the present series of experiments, the most promising compositions were tested both in vitro and in vivo. The materials science part, including mechanical tests, was conducted in our previous work. Tensile tests and compression tests were used to compare the cross-linked material to the original freeze-dried gelatin. Based on the mechanical tests, we selected the material that was optimal, and our results support that BDDE is the most promising cross-linker. The ultimate goal of the present development is to create a scaffold that can be regulated as a medical device. In order to go through the regulatory process, there are applicable standards. For biocompatibility, the ISO 10993 [37] is the applicable standard, which has recommended endpoints, based on the type of medical device, the type of patient contact, and the duration of patient contact. According to ISO 10993 [37], our envisioned device is an “implant device” that is in contact with tissue/bone, and over a period of 30 days, which falls into the category of a permanent implant device [13]. As such, the required tests include cytotoxicity, sensitization, irritation, acute systemic toxicity, subacute toxicity, genotoxicity, and implantation. One of the methods to test toxicity according to the standard is the XTT method; the viable cells indicate that the scaffold is non-toxic.

The number and size of the pores were similar after the cross-linking, which reaffirms that the matrices are suitable for cell seeding [38,39,40]. The SEM images also revealed that the mechanical disadvantages of the scaffold that were produced with the use of DVS are probably due to the small cracks that are also visible on the 50x magnification image, and are in accordance with earlier observations [41] that the structure was generally more rigid, probably due to the more rigid vinyl bonds that are formed between the amino groups and DVS. PEGDE resulted in an uneven surface compared to BDDE. Probably the epoxy bonds and the longer chain present in the cross-linker allow the epoxy cross-linked gelatin to have a more flexible structure, and, as the PEGDE chain is longer compared to the BDDE chain, that is a possible reason why PEGDE can allow a less uniform surface. To elaborate on the results of the cell seeding on the scaffolds, some technological limitations need to be addressed. Probably only a small portion of the 10,000 cells was able to remain on the surface of the 5 mm diameter scaffold, while the rest settled at the bottom of the plate. This likely explains the similarity of the scaffolds compared to the negative control, and the relatively low difference after 168 h, as shown in Figure 3. Thus, the experiment was repeated with 2 cm diameter scaffolds, with the use of 20,000 cells; in this case, the higher surface allowed for a larger success of attachment. In this case, the viability of the seeded cells was significantly higher in all the groups after 168 h of incubation compared to 24 h of incubation, which indicates that the cells were able to attach and proliferate. This was also visualized in a representative fluorescent microscopy image, as shown in the images in Figure 4. In this case, 5 mm diameter cylinders were cut using a biopsy punch from the original 2 cm diameter scaffold to obtain a sample with suitable dimensions and geometry to allow visualization. It is important to point out that hBM-dMSCSs only proliferate until the cells are confluent; thus, the diameter, pore size, and fiber size are crucial to support attachment and proliferation [42], and pre-incubation is also a common step [43]. The XTT measures the viability of the cells. This method produces a chromophore that has the maximum absorbance at 460 nm, and the absorbance is directly proportional to the mitochondrial activity of cells, which is directly proportional to the number of living cells. Thus, it is suitable to both measure the viability of freshly attached cells and the viability of the cells that were allowed to proliferate during the 168 h incubation after seeding. Our development focused on a potentially applicable medical device for implantation. We only used in vitro tests to ensure that the implants can be used safely, and the implants are non-toxic; thus, after we saw that the absorbances increased in all the cases using the 2 cm diameter scaffolds after 168 h of incubation compared to the absorbance after 24 h, we concluded that those cells that were able to attach to the surface were able to proliferate. Based on these findings, BDDE was the chosen cross-linker to test the in vivo compatibility of the cross-linked scaffolds.

No inflammation or total degradation was observed either after one or three months. The explants were observed with the use of optical microscopes to depict the attachment of tissues and the formation of blood vessels. Pro-inflammatory markers were not quantified; we evaluated the potential inflammation by observing cell morphology and tissue response in vivo. H&E and Masson’s trichrome staining revealed no abnormal morphology, immune cell infiltration, or fibrous capsule or giant cell formation, indicating the absence of inflammation. Although the development is still in the scale-up phase, for medical device development, we will need to conduct standardized biocompatibility tests in accordance with ISO 10993 [37], including cytotoxicity, sensitization, and irritation assays. It was observed that the size of the scaffolds did not change after one month or three months, and the attachment of adipose tissue and blood vessel formation inside the scaffold was visible. It is also visible that the inner side of the implants contained more adipose tissue, which is not surprising, taking into account that the vascularization is more effective on the inner side of the implant; thus, that is where the remodeling starts from. The vascularization and the attachment of adipose tissue indicate that the implants are biocompatible, which is advantageous in reconstructive surgery [44,45].

The potential in vivo progress was further analyzed with histological measurements. In the case of EvG staining, there was no significant difference between the relative area of the nuclei, which indicates that the number of cells that were present is similar in each scaffold. However, between the ECM and cellular components, there was a significant difference between Group I and Group II and between Group I and Group III.

Group II had a more alkaline solution and cross-linker during the reaction; thus, it would have been expected that the structure would degrade more easily compared to Groups I and III, which had a less alkaline solution and cross-linker. Given that Group I was implanted for only 4 weeks, compared to Group III, which was implanted for 12 weeks, we would have expected larger differences in the scaffold area and blood vessel formation.

The relative area of the scaffolds did not differ significantly according to the evaluation with the use of three different staining, which led to the conclusion that with our cross-linking method, the scaffolds did not degrade significantly compared to the size of the scaffolds at the time of the implantation. To mention a few, in vivo soft tissue implants for comparison, porous elastin–collagen scaffold was used in a submucosal soft tissue implant, and the collagen part degraded after 15 days, the elastin part persisted up to 90 days [46]. In a thin collagen sheet in rats (subcutaneous implant), the native collagen matrix maintained tissue compatibility and was completely degraded by day 42 [47]. BioGide^®^, a bi-layered collagen membrane, showed gradual resorption: partial at 1–2 months, severe by 3 months, and largely absent around 4 months in a dental application, with moderate vessel formation [48]. A silk fibroin–collagen type II composite cartilage in rabbits degraded gradually after 8 weeks, with fibrous tissue filling after 12 weeks [49]. Our goal was to fabricate a biocompatible scaffold that is non-toxic, non-immunogenic, does not degrade over three months, and enables vessel formation. The envisioned product that we aim to develop is a permanent flexible implant that is regulated as a medical device; thus, the preliminary results seem to support our expectations. The planned product is intended to become a soft tissue implant, and the closest material to the cross-linked gelatin is collagen. Two marketed products are comparable to our proposed product from Allergan (Zyplast and Zyderm), which were acquired from Inamed (formerly known as Cosmoderm and Cosmoplast). All of them were discontinued, and all of them contained collagen, which was bovine-derived. Zyplast contained glutaraldehyde cross-linked collagen [50]. Nowadays, hyaluronic acid compositions are used; however, these products do not support cell attachment, and, generally, slowly degrade over 3–6 months [51]. Our advantage is that the cost of gelatin is less than that of collagen or hyaluronic acid, and we generally aim to have a medical device that can have a pre-defined 3D shape, which becomes an integral part of the body due to remodeling. Along with these advantages, our material is able to withstand heat sterilization and collagenase enzyme-induced degradation, and does not significantly degrade over a period of 3 months.

## 4. Materials and Methods

### 4.1. Scaffold Preparation

Based on our previous results, two epoxy cross-linkers (BDDE and PEGDE, Sigma-Aldrich, St. Louis, MO, USA) and one vinyl cross-linker (DVS, ABCR, Karlsruhe, Germany) were tested. The optimal reaction parameters were used with reduced volume to enable the in vitro and in vivo experiments. Thus, the hydrogel was prepared using 50 mg of gelatin (Gelita AG, Sinsheim, Germany) that was weighed on an analytical balance and dissolved in 1 mL of RO water at 50 °C. The resulting solutions were lyophilized at −55 °C and 5 Pa for 24 h. The whole 1 mL of starting gelatin samples after freeze-drying were put in the freshly prepared cross-linker composition containing either 4, 12, or 20 µL of cross-linker to prepare the matrices (BDDE or DVS, or PEGDE) and 300 µL of 1 *w*/*w* % NaOH. An amount of 40 µL of cross-linker and 600 µL of 1 *w*/*w* % NaOH was also tested with the use of BDDE. The exact preparation method, as part of the optimization process, is indicated in Table 1 for a better understanding.

The reaction mixture was allowed to cross-link for 48 h at 4 °C. The cross-linked gels were washed with 5 mL of RO water thrice, and were heat sterilized. The heat-sterilized scaffolds were cut to 5 mm diameter pieces using a biopsy punch and were freeze-dried again to reach the final form.

### 4.2. Cell Culture

Human bone marrow-derived mesenchymal stem cells (hBM-dMSCs, Sigma-Aldrich, St. Louis, MO, USA) isolated from bone marrow aspirate were cultured in T75 TC-treated culture flasks in an incubator at 37 °C, 5% CO_2_, and 95% humidity. The hBM-dMSCs were maintained in a stem cell medium: Dulbecco’s modified Eagle’s medium (DMEM) containing 4.5 g/L glucose and L-glutamine (Lonza, Basel, Switzerland) supplemented with 10% fetal bovine serum (FBS; EuroClone, Pero, Italy), 1% Penicillin–Streptomycin (Sigma-Aldrich, St. Louis, MO, USA), and 0.75 ng/mL basic fibroblast growth factor (Sigma-Aldrich, St. Louis, MO, USA). The culture medium was refreshed 3 times a week, and all cell culture procedures were carried out in a sterile laminar flow tissue culture hood.

### 4.3. Viability of hBM-dMSCs Cultured on the Scaffolds Using XTT

Two sizes of matrices were tested; the 5 mm diameter pieces were placed into the wells of 96-well, ultra-low attachment plates in 200 µL of stem cell medium, and 10,000 hBM-dMSCs (6 passages) were seeded on each scaffold. The next day, the viability of the seeded cells was measured using Cell Proliferation Kit II (XTT; Roche, Mannheim, Germany), according to the manufacturer’s instructions, on half of the membrane-containing wells to measure the amount of attached cells. The rest of the scaffolds with the seeded cells were cultured for 6 more days in 200 µL of stem cell medium. The medium was refreshed every 2 days. On the 7th day, the viability of the cells on the scaffolds was examined using XTT to compare the proliferation on the scaffolds. For these 5 mm diameter samples, 50 µL XTT was added to 100 µL of stem cell medium, and was incubated for 4 h. The 2 cm diameter pieces were placed into the wells of a 6-well plate. On the first day, 20,000 hBM-dMSCs (6 passages) were seeded onto the matrices on 6-well ultra-low attachment plates in 500 µL of stem cell medium. The medium was refreshed 24 h later with 2 mL of stem cell medium, and the viability of half of the seeded cells was measured; the rest was cultured for 168 h, and the medium was refreshed thrice during the week. On the 7th day, the viability of the cells on the scaffolds was examined using XTT to compare the proliferation on the scaffolds. For these larger samples, 200 µL of XTT was added to 2 mL of stem cell medium, and was incubated for 4 h. The absorbances were measured at 460 nm. The negative control only contained the medium and the reagent, without cells.

### 4.4. Animals and Surgical Procedures

In our experiments, 3–4-month-old C57Bl/6N mice were used. The experiments were carried out according to the guidelines of the Hungarian Law of Animal Protection (XXVIII/1998), and all procedures were approved by the National Scientific Ethical Committee on Animal Experimentation (PEI/001/2706-13/2014, approval date: 17 December 2014, and PE/EA/00487-6/2021, approval date: 30 December 2021). Animals were housed at a constant temperature with a 12 h light/12 h dark cycle, and they had ad libitum access to food and water. Before anesthesia, the weight of the mice was measured, which ranged between 30 and 40 g. Anesthesia was induced with 3% isoflurane, and after placing the mice on a heating pad, the concentration was reduced to 2.5% for the duration of the procedure. The dorsal area of the mice was depilated using hair removal cream at the designated site, followed by cleaning with alcohol. A skin incision of approximately 1 cm was made on the back, and subcutaneous pockets were carefully created along the incision line using fine scissors. The implants were inserted into the prepared pockets, and the incision was closed with 5/0 silk sutures. At the end of the procedure, the surgical site was treated with Betadine and continued to be treated for an additional 3–4 days to promote healing and maintain cleanliness. The mice received antibiotics (Amoxicillin/clavulanic acid, Antapharma 1000 mg/200 mg − 0.05 × mouse weight (g) × 100 µL) intraperitoneally to prevent inflammation and infection. The day after surgery, the mice were returned to their original bedding cages. The implanted scaffolds were all 5 mm in diameter, and all contained 1 mL 5 *w*/*w* % GEL as a starting composition. The difference was in the constituents and the implantation time, as described below in Table 2 (n = 3).

After the applied implantation period (either 1 or 3 months), the animals were sacrificed by cervical dislocation, and their backs were depilated. An incision was made to expose the implants, which were observed by a light microscope (Leica M80; Leica Microsystems, Wetzlar, Germany) and then removed. The procedure was repeated after twelve weeks with the 20 animals in the 12-week group. After the scaffolds were removed, they were fixed in 4% formaldehyde, their weights were measured, and they were sent for histology measurements.

### 4.5. Microscopic Images and Cell Adherence Test by Calcein Staining

The images of the explants were observed using optical microscopes. The used microscopes were either a Leica M80 (Leica Microsystems, Wetzlar, Germany) with 0.75 or 2.5× magnification or a Zeiss AXIO Imager A1 (Carl Zeiss MicroImaging GmbH, Göttingen, Germany) at 2×, 4×, 10×, or 20× magnification, or a digital optical Keyence VHX-5000 microscope, with a VH-Z100R objective, capable of magnifying between 100× and 1000×.

Viable cell adherence was tested using Calcein-AM staining. Cell adherence tests were conducted to investigate if MSCs adhere and proliferate on the crosslinked matrices. Sterile 2 cm diameter pieces were used for cell seeding. On the first day, 20,000 MSCs (6 passages) were seeded onto the gels on 6-well low attachment plates in 500 µL stem cell medium. The medium was refreshed 24 h later with 2 mL of stem cell medium and then three times a week. On the 14th day, 5 mm diameter pieces were cut from the original matrix, and the attached cells were stained. The scaffolds were washed thrice with phosphate-buffered saline (PBS) and stained in PBS containing 1 mM of Calcein-AM (Invitrogen, Carlsbad, CA, USA) and 20 mg/mL of Hoechst (Invitrogen, Carlsbad, CA, USA) for 30 min. The matrices were washed again thrice for 10 min with FluoroBrite DMEM (Gibco, Paisley, Scotland) containing 5% l-glutamine (Sigma Aldrich, St. Louis, MO, USA) and 10% FBS, and images were taken by an inverse fluorescent Nikon Eclipse Ti2 microscope (Nikon Corporation, Tokyo, Japan).

### 4.6. SEM Images

The surface microstructures of the cells that were seeded and cultured on the scaffolds were examined by a scanning electron microscope (SEM) (JEOL JSM-6380LA, Jeol Ltd., Tokyo, Japan). Before microscopic observation, the membranes were fixed with 2.5% glutaraldehyde for 20 min. Dehydration was performed with increasing concentrations of ethanol (50%, 70%, 80%, 90%, 100%) for 5 min each. After dehydration, samples were treated with 0.5 mL of 100% hexamethyldisilazane (HMDS) (Sigma Aldrich, St. Louis, MO, USA) for 5 min, and they were left in the safety cabinet overnight to allow excess HMDS to evaporate. After drying, the samples were sputter-coated with gold (JEOL JFC-1200 Fine Coater, 12 mA, 20 s, Jeol Ltd., Tokyo, Japan) and examined under SEM. The middle region of the surfaces was scanned using 50×, 100×, 250×, 500×, 1000×, 5000×, and 10,000× magnification. The SEM images were used to assess morphological characteristics; the calculation was performed with ImageJ using the 50× magnification images.

### 4.7. Histological Procedures

The specimens were dehydrated in a graded series of alcohol and embedded in polymethylmethacrylate. Slices in the longitudinal direction of the implant were cut with a laser microtome (TissueSurgeon, LLS ROWIAK GmbH, Hannover, Germany) and stained with Hematoxylin and Eosin (HE), Elastica van Gieson (EvG), Masson and Goldner (MG), and Movat’s Pentachrome (MP). Slice thickness was 30 µm. Scanning and digitalizing for evaluation were performed using an optical microscope Zeiss AXIO Imager A1 (Carl Zeiss MicroImaging GmbH, Göttingen, Germany) at 2×, 4×, 10×, or 20× magnification. Samples were evaluated qualitatively in terms of structure and degradation of the scaffold (preserved fiber structure), reaction of surrounding tissue (cell infiltration), and cell migration into scaffolds.

### 4.8. Statistical Analysis

One-way analysis of variance (ANOVA) with Tukey’s post hoc test or two-way ANOVA with Sidak’s multiple comparison test was performed to compare the means of groups using Prism 7 software. The significance level was *p* < 0.05, where * means that *p* is between 0.01 and 0.05, ** means that *p* is between 0.01 and 0.001, *** means that *p* is lower than 0.001, and **** means that *p* is lower than 0.0001, and data are presented as mean ± standard error of the mean, “n” is the number of samples from the same group.

## 5. Conclusions

This study focused on the in vitro cell support and in vivo remodeling of gelatin-based scaffolds, which were found to have optimal mechanical properties. The freeze-dried gelatin matrices were sterile and supported the seeding and proliferation of hBM-dMSCSs in vitro. The surface of the scaffolds showed differences when observed with SEM: the use of DVS led to smaller cracks, the use of PEGDE led to uneven surfaces; thus, BDDE was the best candidate. Based on the in vitro results, BDDE-containing scaffolds were implanted for four weeks or three months in the backs of mice. We found that the implants were biocompatible and did not induce inflammation. The implants enabled the formation of blood vessels and the adherence of adipose tissue, and did not degrade significantly after three months, according to optical microscopic images and histological evaluation. In conclusion, our material appears suitable as a matrix for medical applications, particularly as a medical device, with the intended use of being a soft tissue implant in regenerative medicine.

## Figures and Tables

**Table 1:** Identification of the matrices.

ID	Constituents
50G1B	1 mL 50 mg/mL freeze-dried GEL, 4 µL BDDE, 300 µL 1% NaOH
50G3B	1 mL 50 mg/mL freeze-dried GEL, 12 µL BDDE, 300 µL 1% NaOH
50G5B	1 mL 50 mg/mL freeze-dried GEL, 20 µL BDDE, 300 µL 1% NaOH
50G5BX2	1 mL 50 mg/mL freeze-dried GEL, 40 µL BDDE, 600 µL 1% NaOH
50G1P	1 mL 50 mg/mL freeze-dried GEL, 4 µL PEGDE, 300 µL 1% NaOH
50G3P	1 mL 50 mg/mL freeze-dried GEL, 12 µL PEGDE, 300 µL 1% NaOH
50G5P	1 mL 50 mg/mL freeze-dried GEL, 20 µL PEGDE, 300 µL 1% NaOH
50G5D	1 mL 50 mg/mL freeze-dried GEL, 20 µL DVS, 300 µL 1% NaOH

**Table 2:** Identification of the in vivo implanted scaffolds.

ID	Constituents	Implantation Time
Group I (I)	1 mL 50 mg/mL freeze-dried GEL, 20 µL BDDE, and 300 µL 1 *w*/*w* % NaOH	4 weeks
Group II (II)	1 mL 50 mg/mL freeze-dried GEL, 40 µL BDDE, and 600 µL 1 *w*/*w* % NaOH	4 weeks
Group III (III)	1 mL 50 mg/mL freeze-dried GEL, 20 µL BDDE, and 300 µL 1 *w*/*w* % NaOH	12 weeks

## Data Availability

The data presented in this study are available on request from the corresponding author.
